# Save Antibiotics: a call for action of the World Alliance Against Antibiotic Resistance (WAAAR)

**DOI:** 10.1186/1471-2334-14-436

**Published:** 2014-11-28

**Authors:** Jean Carlet, Claude Rambaud, Céline Pulcini

**Affiliations:** Intensive care specialist, 9 rue de la Terrasse, 94000 Creteil, France; Comité inter-associatif pour la santé »(CISS), Paris, France; CHU Nancy, Service de Maladies Infectieuses, Nancy, France; Université de Lorraine, EA 4360 Apemac, Nancy, France; World Alliance Against Antimicrobial Resistance (WAAAR), Paris, France

**Keywords:** Antibiotic resistance, New antibiotics,alliance

## Summary

Resistance to antibiotics has recently increased dramatically worldwide. The pipeline of new classes of antibiotics is dry for at least the next few years. Therefore antibiotic resistance represents one of the most problematic public health issues of our time. Treatment failures already happen in increasing numbers for common community-acquired infections, such as urinary tract infections or intra-abdominal infections. This is due in particular to Enterobacteriaceae harboring extended-spectrum beta-lactamases (ESBL). Enterobacteriaceae harboring carbapenemases are also highly prevalent in many countries. In the future, difficult surgical procedures, transplants, and other immunosuppressive therapies may become very risky. Resistance is mainly due to an excessive usage of antibiotics, in both humans and animals, and to cross-transmission of resistant bacteria. Action is urgently needed. Therefore, a World Alliance Against Antibiotic Resistance (WAAAR) was created in 2011. It includes healthcare professionals, consumers, health managers, and politicians. We present here the main measures proposed by the Alliance, as a result of a strong consensus between the different stakeholders, including general practitioners and veterinarians.

Antibiotics are in great danger [1]. Antibiotic resistance has increased dramatically in the last few years, and very few new compounds have been marketed in the recent years, and will be made available in the next few years [2]. Therefore, antibiotic resistance represents one of the most important public health issues of our time [3]. Meticillin-resistant *Staphylococcus aureus* (MRSA) infections, in particular due to community-acquired MRSA [4], are extremely prevalent in many countries, including some European ones (European Center for Diseases Control and Prevention. EARSS-Net database. http://ecdc.europa.eu) [5], the USA, South America and Asia. Prevalence of MRSA infections decreased in the last few years in many European countries, and this can be considered as a very positive and promising result. Vancomycin-resistant enterococci (VRE) are also very frequent, with large differences between countries. In the European point prevalence study organized by ECDC [6], VRE prevalence was going from 1% to more than 50%. The prevalence of *Escherichia coli* and *Klebsiella pneumoniae* harboring extended-spectrum beta lactamases (ESBL) is increasing regularly worldwide [7] reaching 50 to 70% for *Escherichia coli* in some European or Asian countries [5, 7]. In the European point prevalence study, prevalence of Klebsiella pneumoniae with carbapenemases was going from 1% to more than 50% [6]. Because they are worried by possible failures for their patients due to those resistant bacteria, clinicians overuse carbapenems, our last line of therapy, to treat common community infections like urinary tract infections or intra-abdominal infections. As a consequence, carbapenemases are more and more frequent in Enterobacteriaceae, *Pseudomonas aeruginosa* and Acinetobacter spp. The prevalence of those multi-resistant strains is very high in several countries like Greece, Turkey, Italy, North African countries and Asian countries, in particular India [8]. People who come back from those countries can carry those resistant strains in their digestive tract and can transmit those strains to other patients in the hospital or to their relatives in the community [9]. If the carriage is not detected early enough in the hospitals, this can lead to outbreaks. Extensively resistant, or even pan-resistant strains are more and more common, in particular in the ICU [10, 11]. Old drugs like colistin have to be used more and more frequently, and strains resistant to colistin have been regularly described over the past few years [12]. Morbidity and mortality due to antibiotic resistance are substantial. It is estimated that 25.000 patients in Europe, and 23.000 patients in the USA die every year from infections due to multi-resistant bacteria. The cost induced by antibiotic resistance is 1.5 Billion Euros (European Centre for Infection Control and Prevention and European Medicines Agency release 2009). In the future, difficult surgical procedures, transplants, and other immunosuppressive therapies may become very risky. Resistance is due to many factors, in particular the overuse of antibiotics in both humans and animals, and the cross-transmission of resistant micro-organisms in both the community, the hospitals and the livestocks.

Antibiotics are very special drugs because their target is a living one, able to adapt and become resistant to the drug. This is very unique. In addition, the effect of antibiotics is not only visible in the treated patients, but also in other patients, since antibiotics act not only on the micro-organism(s) responsible for the treated infection, but also on the commensal flora. The gut is the silent epicentre of antibiotic resistance, because the antibiotics modify profoundly the gut microbiome, and allow resistant micro-organisms to grow and to colonize this organ for prolonged periods of time [9]. Those resistant strains can then be transferred to other patients in the hospitals, or to relatives in the community. Antibiotics, and resistant micro-organisms present in the effluents can contaminate the environment [13]. Micro-organisms carried by animals can contaminate humans via either the environment or the food chain [14].

Antibiotics are overused nearly everywhere. There are huge differences in their usage between countries. For example, Scandinavian countries use one third of the amount used by countries like France and Greece [15]. There is a clear relationship between the consumption of antibiotics and the resistance level [16]. It is more than unlikely that Scandinavian patients are less well treated than patients in France or Greece. In fact, life expectancy is even longer in Scandinavia than in many other countries. It is known that in certain countries between one third and half of the antibiotic therapies are either unnecessary or inappropriate, both in the in- and the outpatient settings. Patients are very often treated with antibiotics for viral diseases, in particular for upper tract respiratory infections, or for simple colonization, in particular for asymptomatic bacteriuria. Even when the treatment is indicated, patients are often treated for too long periods of time.

It is really time to act vigorously in order to save antibiotics through an active protection of available antibiotics and the acceleration of the innovation to bring new drugs to the clinicians in the near future [17, 18]. The action must be global and worldwide.

WAAAR (World Alliance Against Antibiotic Resistance) is a cross-cutting action plan designed by a group of professionals and by the consumers’ group LIEN to deal with the current emergency. This plan involves healthcare providers (both in the in- and outpatient settings), veterinary medicine professionals, politicians and consumers. The problem of antibiotic resistance is of immediate significance for current and future users of the healthcare system, as well as for all of us in the general population. WAAAR’s main objective is to raise awareness regarding this public health concern, among politicians, public health authorities, international agencies, health care professionals, patients, and the entire population, who represent the target of our action.

The World Alliance Against Antibiotic Resistance is a group of about 700 individuals representing all the key stakeholders including healthcare system users: “Infections nosocomiales, sécurité des soins, accompagnement des maladies” (LIEN), Collectif inter-associatif pour la santé (CISS), Patients for Patient Safety [WHO], and the Association for the Defense of Victims of Nosocomial Infections [ADVIN] in Quebec). People are coming from 55 different countries. The Alliance receives support from 85 learned societies and 55 various professional groups in France and throughout the world. It is a nonprofit organization (French law of 1901) open to professionals and users worldwide. The alliance is not sponsored by the pharmaceutical industry.

We present here the main measures advocated by WAAAR to curb antibiotic resistance:

Antibiotics must be actively protected like as a precious resource [17]. Antibiotic prescription is still considered everywhere like a trivial act. In many countries antibiotics are available “over the counter”. This must be combated, as well as the use of counterfeit or outdated antibiotics. Antibiotics are widely available and wasted in developed countries, but the access to those drugs is limited in many developing ones [19]. Like for water and food, this is not acceptable.

Antibiotic stewardship programs must be implemented everywhere [20]. Again, antibiotics being very special drugs, special ways to prescribe antibiotics must be implemented. They must be prescribed on dedicated nominative forms. For some antibiotics, carefully listed in every hospital, a prior authorization from an infectious diseases (ID) specialist must be organized. This must be the case for example for carbapenems, penicillins associated with beta-lactamases inhibitors, colistin, tigecyclin, glycopeptides, daptomycin, or oxazolidinones. A systematic re-evaluation must be scheduled around day 3 of therapy [21]. At this stage, a de-escalation is frequently possible. It is more difficult to implement this re-evaluation in primary care, but the concept is still valid for most infections. Duration of therapy must be carefully determined, be as short as possible and mentioned on the very initial prescription. PK/PD considerations are also very important issues. It is very frequent to use too low dosages of antibiotics, in particular in obese patients, or in severely ill patients in the intensive care units (ICU). In some cases antibiotic blood levels must be monitored. Finally, both in the community and the hospitals,in humans and animals, the use of cephalosporins and fluoroquinolones must be limited.

Infection control measures are essential. The prevention of cross-transmission of multi-resistant bacteria both in hospitals, the community, and livestock is of paramount importance. Hand hygiene is the most important action. Isolation procedures are also important, as well as the whole body disinfection with chlorhexidine in patients in the ICU [22].

Environmental measures are also of paramount importance. Sanitation problems are very problematic in many countries. Everything must be done to treat used water, in particular when coming from the hospitals or the farming facilities.

Antibiotics or treatment strategies for veterinary medicine that have the smallest possible ecological impact must be promoted. A ban on the use of antibiotics as growth factors, a ban on their prophylactic use, the limitation of the use of antibiotics critical for humans, like cephalosporins and fluoroquinolones are important points.

The development of new antibiotics needs the implementation of innovative measures (cooperation between companies, private/public cooperation, increase in the duration of the patent of the drugs, financial reward, orphan drugs-like programs….). A user fee in veterinary medicine has been recently advocated [23].

The expansion of basic and applied research efforts in human and veterinary medicine is key. Large budgets have been allocated in the past few years, particularly by the NIH or the European commission, but more resources are needed.

Available vaccines (pneumococcus, influenza virus…) must be widely used. New vaccines must be developed. Alternatives to antibiotics are also a promising strategy (drugs acting upon virulence and upon quorum sensing for example).

A widespread use of rapid diagnostic tests, with the goal of limiting the prescription of antibiotics to proven bacterial infections only, is needed. New tests need to be developed, and the use of existing tests should be promoted. As an example, in France, rapid antigen diagnostic tests for acute pharyngitis are underused by general practitioners, although they are freely available [24].

A surveillance of antibiotic resistance and use, with regular feedback to healthcare professionals and the public is necessary. There are efficient networks in certain countries and continents [5, 15], but they are lacking in most countries.

Education and training programs for healthcare professionals and consumers are of considerable importance [25]. Large information campaigns have been shown to be very efficient [26].

Safeguarding antibiotics requires an orchestrated effort carried out jointly by healthcare system users and prescribers (in the broad sense of the term) [27, 28]. Therefore, WAAAR has chosen as its primary objective to raise awareness among all stakeholders of the urgency and magnitude of the threat. The Alliance must lobby actively for antibiotics beyond the circle of insiders in order to raise awareness among policy-makers, international health organizations (World Health Organization (WHO), World Organization for Animal Health (OIE), Food and Agriculture Organization of the united Nations (FAO), European Centre for Disease Prevention and Control….), and the entire population. WAAAR works with other non-governmental organizations like the Alliance for the Prudent Use of Antibiotics (APUA), the Global Antibiotic Resistance Partnership (GARP),the Action on Antibiotic Resistance (REACT) and Antibiotic Action. WAAAR intends to ask the UNESCO to put the “concept of antibiotics” on the list of the immaterial world heritage.

- The Alliance intends to launch a solemn declaration against antibiotic resistance. This document will be widely promoted, and published in many different journals worldwide.
